# Ferroelectricity‐Enhanced Piezo‐Phototronic Effect in 2D V‐Doped ZnO Nanosheets

**DOI:** 10.1002/advs.201900314

**Published:** 2019-06-22

**Authors:** Yejing Dai, Changsheng Wu, Zhiyi Wu, Zhihao Zhao, Li Li, Yang Lu, Zhong Lin Wang

**Affiliations:** ^1^ School of Materials Sun Yat‐sen University Guangzhou 510275 China; ^2^ School of Materials Science and Engineering Georgia Institute of Technology Atlanta GA 30332‐0245 USA; ^3^ Key Laboratory of Advanced Ceramics and Machining Technology Ministry of Education School of Materials Science and Engineering Tianjin University Tianjin 300072 China; ^4^ Beijing Institute of Nanoenergy and Nanosystems Chinese Academy of Sciences Beijing 100083 China

**Keywords:** coupling effect, ferroelectricity, photodetectors, piezo‐phototronic effect, ZnO nanosheets

## Abstract

Emerging 2D electronic materials have shown great potential for regulating and controlling optoelectronic processes. A 2D ferroelectric semiconductor coupled with the piezo‐phototronic effect may bring unprecedented functional characteristics. Here, a heterojunction photodetector made of p‐Si/V‐doped‐ferroelectric‐ZnO 2D nanosheets (FESZ‐PD) is fabricated, and the ferroelectricity‐enhanced piezo‐phototronic effect on the photoresponse behavior of the FESZ‐PD is carefully investigated. By introducing the ferroelectricity and the piezo‐phototronic effect, improved current rectification performance is achieved and the photoresponse performance of the heterojunction is enhanced in a broad spectral range. The applied voltage bias during measurement naturally causes ferroelectric spontaneous polarizations to align, resulting in a change in band structure near the interface and the local piezo‐phototronic effect. The modulated energy band promotes the generation, separation, and transportation efficiency of photogenerated carriers greatly. Compared with the Si/ZnO 2D nanosheets photodetector without ferroelectricity under strain‐free conditions, the photoresponsivity *R* of the FESZ‐PD increases by 2.4 times when applying a −0.20‰ compressive strain at +1 V forward bias. These results confirm the feasibility of coupling the ferroelectricity with the piezo‐phototronic effect in 2D ferroelectric materials to enhance the photoresponse behavior, which provides a good way to enable the development of high‐performance electronic and optoelectronic devices.

Wurtzite ZnO nanostructures have attracted widespread attention due to their unique physical properties, such as optical, semiconducting, piezoelectric, and pyroelectric properties.^1^, ^2^, ^3^, ^4^ Among them, 2D ZnO nanosheets (NSs) with high‐surface area and large planar dimensions are of great interest and have emerging applications in catalysis and electronics field.^5^, ^6^, ^7^, ^8^ However, despite many interests in 2D nanomaterials, the preparation and utilization of ferroelectric ZnO 2D nanostructure‐based electronic devices have rarely been investigated except for a few applications in nanogenerators.^9^, ^10^ Ferroelectric materials, as a special type of piezoelectric materials with spontaneous polarizations that can be switched easily by an externally applied electric field, have shown large potential to modulate the electronic and optoelectronic process and thus have led to many exciting research fields.^11^, ^12^ For example, ferroelectric polarizations have been extensively utilized to tune the transport properties of the field‐effect transistors, tunnel junctions, and photovoltaics;^13^, ^14^, ^15^, ^16^ however, few of them are related to the inorganic ferroelectric semiconducting nanomaterials. Therefore, it is of great significance to explore new applications of ferroelectric ZnO 2D NSs in optoelectronic devices.

In recent years, the piezo‐phototronic effect, originated from the strain‐induced piezoelectric polarization charges in some non‐centrosymmetric piezoelectric semiconductors (CdS, MoS_2_, ZnO, InN, etc.) and capable of tuning/modulating the charge carrier transport behavior (e.g., generation, separation, and recombination) near the junction or interface during the optoelectronic process, has been proved to be an effective means to enhance the optoelectronic performances.^17^, ^18^ Therefore, the piezo‐phototronic effect has attracted great attention recently and has been extensively utilized in photovoltaics, light emitting diodes, and photodetectors (PDs).^19^, ^20^, ^21^ However, most of the researches focused on the piezoelectric but nonferroelectric semiconductor materials, which limits the application of the piezo‐phototronic effect in optoelectronic devices since we need to apply a stress along a certain direction of piezoelectric crystals. In order to utilize the piezo‐phototronic effect more conveniently and efficiently, it is important to reveal the effect of ferroelectric semiconductor on the optoelectronic process of PD devices.

Here, we propose a heterojunction PD made of p‐Si/V‐doped‐ferroelectric‐ZnO 2D NSs (FESZ‐PD) and investigate how the spontaneous polarizations derived from the V‐doped ZnO ferroelectric semiconductor modulate the carrier transport behavior during the optoelectronic process. By introducing the ferroelectricity, the spontaneous electric dipoles that can be switched by the external electric field could modulate the energy band structure near the heterojunction interface, and the realigned energy band promotes the generation, separation, and transportation of photogenerated electron–hole pairs. Under a +1 V forward bias and strain‐free condition, compared to the p‐Si/ZnO 2D NSs photodetector (SZ‐PD) without ferroelectricity, the photoresponsivity *R* of the FESZ‐PD is improved from 35.1 to 61.6 mA W^−1^. Furthermore, the enhanced ferroelectricity caused by the applied static compressive strain could further improve the photoresponse performance, and the photoresponsivity *R* increases to 120.3 mA W^−1^ with faster response speed. The corresponding working mechanism of how the ferroelectricity‐enhanced piezo‐phototronic effect influences the photoresponse performance of the FESZ‐PD is carefully analyzed by investigating the energy band structure change under forward bias and compressive strain conditions. Our results present the feasibility of coupling the ferroelectricity with the piezo‐phototronic effect in 2D ferroelectric materials to enhance the photoresponse behavior, which offers a good route and perspective to enable the development of high‐performance electronic and optoelectronic devices.

The device structure of the FESZ‐PD is shown in **Figure** **1**a. First, in order to increase the photosensing area, micropyramids with a bottom edge length of 3–10 µm (Figure S1a, Supporting Information) are produced on a Si wafer surface through a wet chemical etching method with a 5 wt% KOH solution. After depositing a ZnO seed layer by spin coating, high‐dense V‐doped ZnO 2D NSs, connected with each other, are uniformly synthesized and vertically grown on the surface of the Si substrate through a hydrothermal method (Figure 1b). Due to the addition of V_2_O_5_, the negatively charged VO_2_(OH)_2_^−^ ion would react with the positively charged Zn^2+^‐terminated (0001) polar crystal plane instead of other nonpolar crystal planes, thus effectively hindering the ZnO growth along *c*‐axis direction,^9^, ^22^ which is different from the V‐doped ZnO nanocrystals prepared by the sol–gel method.^23^, ^24^ Therefore, 2D lateral growth of ZnO could be achieved using the inhibition effect of VO_2_(OH)_2_^−^ ions on (0001) crystal plane growth, and the formation of V‐doped ZnO 2D NSs with an average dimensions of about ≈1 × 1 µm (Figure S1b, Supporting Information) and an average thickness of 15–20 nm takes place (Figure 1c and Figure S2a, Supporting Information). The obtained V‐doped ZnO 2D NS shows relatively good crystallization and grows in a vertical [0001] direction (i.e., the (0001) crystal plane is vertical to the silicon surface.), verified by select area electron diffraction (SAED) (Figure 1d). The measured d‐spacing value from Figure S2b in the Supporting Information is found to be 0.28 nm, corresponding to the interplanar spacing of {10–10} planes in wurtzite ZnO crystal. The energy dispersive X‐ray (EDX) spectrum (Figure 1e and Figure S3, Supporting Information) also confirms the successful doping of V element into ZnO with the atomic ratio of Zn/V ≈4.3 and the V‐doped ZnO NSs exhibit good transmission from visible to near‐infrared waveband (Figure 1g). Finally, an indium tin oxide (ITO) layer as top electrode is deposited on the ZnO NSs (Figure S1c,d, Supporting Information) and an aluminum (Al) layer as bottom electrode is deposited on the Si substrate. Moreover, by replacing the V‐doped‐ferroelectric‐ZnO 2D NSs with nonferroelectric‐ZnO 2D NSs (Figure S4, Supporting Information), a reference SZ‐PD device, with the same structure is prepared for comparison. For the nonferroelectric‐ZnO 2D NSs, an Al‐based seed layer is used, and the Al(OH)_4_^−^ also hinders the ZnO growth along *c*‐axis direction and facilitates the 2D lateral growth of ZnO.^25^ In order to ensure the microstructural consistency for the two kinds of ZnO NSs, the same growth solution and growth conditions are used. Detailed preparation process of the FESZ‐PD and SZ‐PD can be found in the Experimental Section.

**Figure 1:**
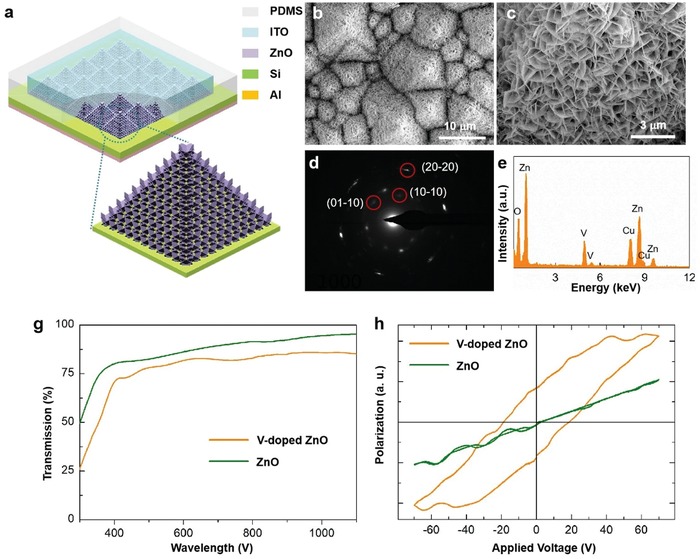
Device structure and characterizations. a) Schematic structure of the FESZ‐PD. b,c) Scanning electron microscopy images of the V‐doped ZnO 2D NSs synthesized on the etched Si wafer. d) Corresponding select area electron diffraction pattern of a ZnO NS. e) Energy dispersive X‐ray spectrum of ZnO NSs. g) Transmission spectra of ZnO NSs and V‐doped ZnO NSs grown on the F‐doped tin oxide (FTO)/glass substrate. h) Polarization hysteresis loops versus electric field (*P–E* loops) of ZnO NSs and V‐doped ZnO NSs grown on a Cu substrate.

By doping V into ZnO, ferroelectricity can be achieved in the V‐doped ZnO 2D NSs owing to the big difference in valence and ionic radii (Zn^2+^ (0.74 Å) and V^5+^ (0.495 Å)) and the randomly oriented electric dipoles in V‐doped ZnO 2D NSs can be easily switched to the direction of the external electric field.^9^, ^26^ In order to measure the ferroelectric property, ZnO NSs and V‐doped ZnO NSs are vertically grown on a copper (Cu) substrate with gold (Au) as top electrode, respectively. As shown in Figure 1h, the ferroelectric polarization hysteresis loop of the ZnO NSs presents a typical paraelectric phase behavior without any remanent polarization, whereas the ferroelectric polarization hysteresis loop dramatically changes by the V doping, confirming the presence of the ferroelectricity due to the remanent polarization.^27^ In addition, it is worth noting that the coercive field and polarization obtained from the polarization hysteresis loop versus electric field (*P–E* loop) of the V‐doped ZnO NSs do not mean their real values. On one hand, the layer of the V‐doped ZnO NSs is not a uniform and smooth film, with various thicknesses (Figure 1c). It is hard to calculate the area value of the electrode, and thus we use *a.u*. as the unit of polarization. On the other hand, some factors, such as the probe contact resistance during the *P–E* loop measurement, would have effect on these values. The actual coercive field is about ≈1 V for the V‐doped ZnO NSs.^9^ Therefore, the external electric field could switch the ferroelectric polarizations under 1 V bias.

The strain‐modulated piezo‐phototronic effect on the heterojunction PD devices was investigated using a homemade experimental setup, as schematically shown in **Figure** **2**a. The PD device is fixed on a sample holder. A fine and stiff rod made of steel is fixed on a stationary XYZ linear translation stage (movement resolution ≈10 µm, 462‐XYZ‐M, Newport Inc.) for facile application of compressive stress on the PD device through a piece of sapphire. In order to compare their photoresponse performance, current–voltage (*I–V*) characteristics of the SZ‐PD and FESZ‐PD were measured, respectively (Figure 2b,c). Under −0‰ and −0.20‰ externally applied static compressive strains, *I–V* characteristics for the SZ‐PD without and with 442 nm light illumination at a power density of 10 mW cm^−2^ were measured and shown in Figure 2b, respectively. The calculation method for external applied compressive strain is the same as that reported in our previous researches.^28^ At +1 V forward bias, the strain‐free SZ‐PD device shows a tiny dark current of 4.9 µA and a photocurrent (*I*_photo_ = *I*_light_ − *I*_dark_, the *I*_light_ and *I*_dark_ are the output current under light illumination and in dark, respectively) of 28.5 µA. When −0.20‰ strain is applied, an increased dark current of 5.7 µA and an increased photocurrent of 44.7 µA are obtained, indicating an improved photoresponse performance due to the piezo‐phototronic effect. Figure 2c shows the photoresponse behavior with increasing light power density under strain‐free condition for the FESZ‐PD device, and the photocurrent displays an approximate linear dependency on the light power density in the measured range (Figure 2d). In addition, the rectification ratio of the heterojunction rises from 2.5 (SZ‐PD under strain‐free condition) to 20.8 (FESZ‐PD under strain‐free condition) under dark condition when a 1 V bias is applied, indicating the improved charge carrier separation efficiency of the p–n heterojunction. Moreover, the photocurrent of the FESZ‐PD reaches to 50.1 µA under 10 mW cm^−2^ 442 nm light illumination, which are larger than that of the SZ‐PD under −0‰ strain (indicated by green triangle in Figure 2d) and −0.20‰ strain (indicated by green star in Figure 2d). These could be attributed to the presence of the ferroelectricity in V‐doped ZnO 2D NSs, which could greatly change the charge carrier transport behavior near the heterojunction. Furthermore, similar results are also obtained under 1064 nm light illumination for the SZ‐PD and FESZ‐PD devices (Figure S5, Supporting Information), confirming the influence of ferroelectricity in a broad spectral range.

**Figure 2:**
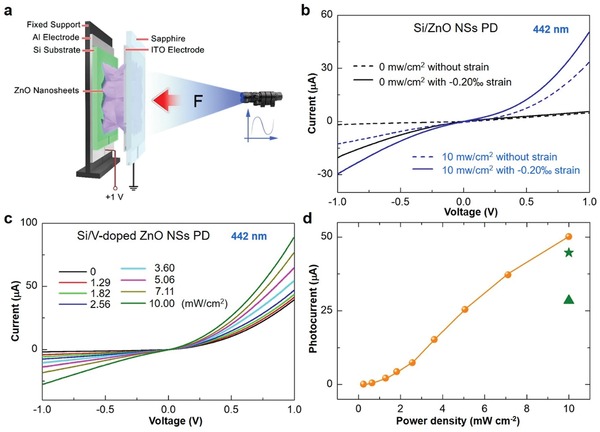
Improved photoresponse performance by the ferroelectricity. a) Schematic diagram of experimental setup for the heterojunction PD devices. b) *I–V* characteristics of the SZ‐PD without (dotted line) and with (straight line) a −0.20‰ compressive strain under dark and light illumination (442 nm at a power density of 10 mW cm^−2^) conditions when a 1 V bias is applied. c) *I–V* characteristics of the FESZ‐PD under different 442 nm light illumination power densities when a 1 V bias is applied. d) Photocurrent changes with the power density at +1 V forward bias (orange ball is for FESZ‐PD, and green triangle and green star are for SZ‐PD without and with −0.20‰ strain, respectively).

In order to investigate the ferroelectricity‐enhanced piezo‐phototronic effect on photoresponse performances of the FESZ‐PD device under different light illuminations (442 and 1064 nm), the photoresponse properties were measured systematically under different static compressive strain conditions. Under different compressive strains, the photoresponse performances of the FESZ‐PD under 442 nm light illumination with three different light power densities (0, 1.29, and 10 mW cm^−2^) are shown in **Figure** **3**a–c, respectively. At +1 V forward bias, the output current gradually increases with the applied compressive strain, as shown in Figure 3d–f. The photocurrents are calculated and presented for various power densities (from 1.29 to 10 mW cm^−2^) under different compressive strain conditions in Figure 3g. With increasing strain, the *I*_photo_ gradually rises (e.g., *I*_photo_ = 50.1 µA under strain‐free condition and *I*_photo_ = 96.2 µA under −0.20‰ strain condition at a power density of 10 mW cm^−2^), indicating the significantly enhanced photoresponse performance of the FESZ‐PD under 442 nm light illumination by the piezo‐phototronic effect.

**Figure 3:**
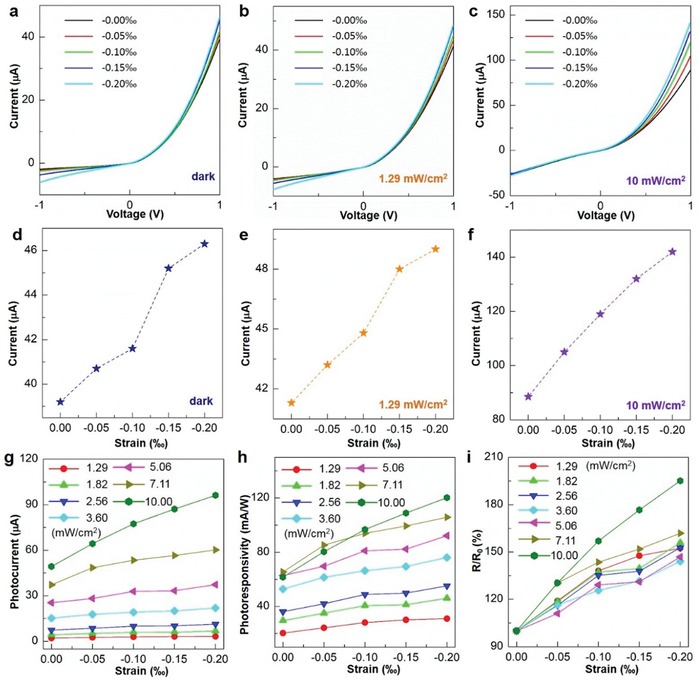
The piezo‐phototronic effect on the FESZ‐PD. a–c) *I–V* characteristics of the device under different compressive strains and 442 nm light illumination conditions when a 1 V bias is applied. d–f) Output currents of the device under different compressive strain and light illumination conditions when the forward bias is +1 V. Strain dependence of g) photocurrent, h) photoresponsivity, i) and *R*/*R*_0_ for the device under different light illumination conditions when the forward bias is +1 V.

The photoresponsivity *R*, an important parameter for PDs, is calculated and plotted in Figure 3h for all power densities with different compressive strains at +1 V forward bias. The *R* is defined as *R* = *I*_photo_ */P*_ill_, where *P*_ill_ is the light illumination power. The *R* value of the FESZ‐PD also increases upon increasing compressive strain. For example, at a power density of 10 mW cm^−2^, the *R* could achieve ≈120.3 mA W^−1^ under −0.20‰ strain, which is near twofold of that (≈61.6 mA W^−1^) under strain‐free condition. The relative change of *R*, defined as *R*/*R*_0_ × 100% (*R* is the photoresponsivity under a certain strain and *R*_0_ is the photoresponsivity under strain‐free condition), is shown in Figure 3i. With increasing compressive strain under a certain power density light illumination, the relative change of *R* gradually increases, showing a large enhancement of photoresponsivity by the piezo‐phototronic effect. The maximum enhancement of *R* could reach ≈195% under −0.20‰ strain at a power density of 10 mW cm^−2^. Moreover, compared with the SZ‐PD under strain‐free condition and −0.20‰ strain condition, the *R* value of the FESZ‐PD under −0.20‰ strain condition is enhanced by 2.4 times and 1.2 times, respectively, owing to the ferroelectricity‐enhanced piezo‐phototronic effect.

Moreover, the current–time (*I–t*) response of the FESZ‐PD device at 10 mW cm^−2^ 442 light illumination under different compressive strains at +1 V forward bias are recorded and shown in **Figure** **4**a. The output currents display obvious switching behavior under various compressive strain conditions ranging from −0.00‰ to −0.20‰, and the repeatability is retained well. The maximum output current increases as the compressive strain increases. The average rise time and fall time are defined as the time interval for the normalized output current rising from 10% to 90% and decaying from 90% to 10%,^29^, ^30^ respectively. An average rise time of 3.43 ms and a fall time of 3.45 ms under strain‐free condition are obtained, respectively. By introducing the piezo‐phototronic effect, an improved response speed is achieved (Figure 4b). With increasing strain from −0.00‰ to −0.20‰, the rise time decreases from 3.43 to 3.07 ms, and the fall time decreases from 3.45 to 3.22 ms. The slightly drop of the rise time and fall time presents better photoresponse performance of the FESZ‐PD under compressive strain condition. Similar results for 1064 nm light illumination are shown in Figures S6 and S7 in the Supporting Information, further demonstrating the greatly improved photoresponse performance by the ferroelectricity‐enhanced piezo‐phototronic effect in a broad spectral region from visible to near‐infrared. The achieved *R* values are competitive with those of the commercial Si photodiode PDs near 442 nm wavelength (0.1–0.2 A W^−1^)^31^, ^32^ and most Si/semiconductor PDs in the visible range (Table S1, Supporting Information), and much larger than those of the commercial Si photodiode PDs near 1064 nm wavelength (<0.1 A W^−1^)^32^ and the Si/semiconductor PDs in the near infrared range (Table S1, Supporting Information).

**Figure 4:**
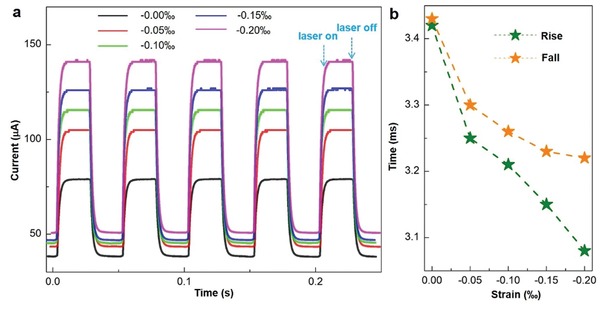
Repeatability and response speed of the FESZ‐PD. a) Transient *I–t* characteristics under different compressive strains at 10 mW cm^−2^ 442 nm light illumination when the forward bias is 1 V. b) Corresponding rise and fall times under 10 mW cm^−2^ 442 nm light illumination and different compressive strains.

In order to explain the working mechanism of the ferroelectricity‐induced improved photoresponse performances as well as the ferroelectricity‐enhanced piezo‐phototronic effect, the energy band diagrams of the FESZ‐PD under different conditions are depicted and analyzed in **Figure** **5** based on Anderson's model.^33^ The bandgap and electron affinity values of ZnO and Si are *E*_g,ZnO_ = 3.37 eV, χ_ZnO_ = 4.50 eV, and *E*_g,Si_ = 1.12 eV, χ_Si_ = 4.05 eV,^21^ respectively. When a Si/ZnO heterojunction is formed, a conduction band offset Δ*E*_c_ = 0.45 eV and a valence band offset Δ*E*_v_ = 2.70 eV are formed at the local interface of p‐Si and n‐ZnO, as shown in Figure 5a (bottom panel).

**Figure 5:**
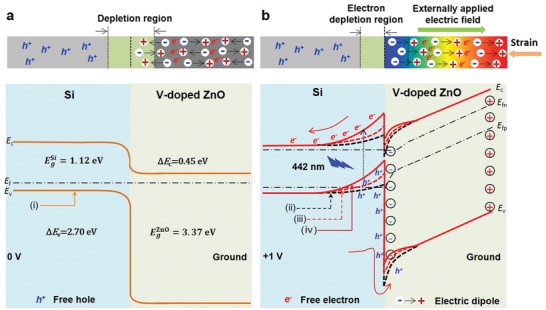
Working mechanism of the ferroelectricity‐enhanced piezo‐phototronic effect. The applied electric field during device measurements naturally causes ferroelectric polarizations aligned, resulting in a change in band structure near the interface and the local piezo‐phototronic effect. a) Schematic band diagrams of the FESZ‐PD without a compressive strain under 0 V bias. b) Schematic band diagrams of the FESZ‐PD with applied compressive strain at +1 V forward bias. The orange straight line, black dashed line, red dashed line, and red straight line respectively represents the energy band under different conditions: i) at 0 V bias with randomly oriented electric dipoles under strain‐free condition, ii) at +1 V forward bias with randomly oriented electric dipoles under strain‐free condition, iii) at +1 V forward bias with aligned electric dipoles under strain‐free condition, and IV) at +1 V forward bias with aligned electric dipoles under compressive strain condition.

When the FESZ‐PD is at 0 V bias under strain‐free condition, a depletion region would be formed at the local Si/ZnO interface and spontaneous electric dipoles randomly orient in V‐doped ZnO, as shown in Figure 5a (top panel). When the FESZ‐PD under strain‐free condition is at +1 V forward bias, two things happen. One is that the energy bands (*E*_c_ and *E*_v_) at the p‐Si side near the local interface change from downward to upward (black dash line in Figure 5b bottom panel),^34^ indicating that an electron depletion region is formed at the Si side and a hole depletion region is formed at the ZnO side near the local interface. The photo‐generated electrons and holes are separated and collected by the Al and ITO electrodes at +1 V forward bias, respectively, forming the photocurrent. Because the probability of holes reaching the ZnO side is small due to the large energy barrier (>2 eV), the photocurrent is mainly from the contribution of photogenerated electrons at +1 V forward bias. The other one is that the electric dipoles in the V‐doped‐ferroelectric‐ZnO 2D NSs would be aligned with the direction of externally applied electric field and the negative ferroelectric spontaneous polarization charges (ferro‐charges) are generated near the pn junction at +1 V forward bias, as shown in Figure 5b (top panel). This is equivalent to forming a build‐in electric field in the FESZ‐PD device, leading to a rise in output current and an enhancement in response speed. Moreover, due to the presence of ferroelectricity‐induced negative ferro‐charges near the interface, the free electrons in the ZnO side would be repelled and the free holes at the Si side would be attracted, resulting in a further upward bending of the energy band for both Si and ZnO sides^17^ and an enlarged electron depletion region in the Si side (red dash line in Figure 5b bottom panel) when compared with the SZ‐PD without ferroelectricity. Considering that the photo‐generated carriers are produced mainly in Si substrate under 442 and 1064 nm light illuminations, hence, the FESZ‐PD has larger photoresponse (*R ≈* 61.6 mA W^−1^) than that (*R ≈* 35.1 mA W^−1^) of the SZ‐PD under strain‐free condition, because the further upward energy band and enlarged depletion region are more conducive to the generation, separation and transportation of photogenerated electrons.

Furthermore, when the external compressive strain is applied, the polarity of the electric dipoles in ferroelectric ZnO will be further enhanced due to the enhanced electronic displacement polarization, leading to a further upward bending of the energy band for both Si and ZnO sides (red straight line in Figure 5b bottom panel).^35^, ^36^ Therefore, as the external compressive strain increases, the photoresponse performance of the FESZ‐PD device is further improved (*R ≈* 120.3 mA W^−1^) due to the piezo‐phototronic effect, and is larger than that (*R ≈* 55.1 mA W^−1^) of the SZ‐PD under the same compressive strain condition, which is attributed to the significantly improved piezoelectricity in V‐doped‐ferroelectric‐ZnO NSs with larger polarity when compared with the nonferroelectric‐ZnO NSs.

In summary, a heterojunction PD made of p‐Si/V‐doped‐ferroelectric‐ZnO 2D NSs has been prepared, and the photoresponse performances with the coupling of the piezo‐phototronic effect and the ferroelectricity under 442 and 1064 nm light illuminations are systematically investigated. By introducing the ferroelectricity, the spontaneous electric dipoles derived from the V‐doped‐ferroelectric‐ZnO 2D NSs can modulate the local band profile near the heterojunction interface, which promotes the generation, separation, and transportation efficiency of photogenerated electron–hole pairs greatly. Furthermore, the enhanced ferroelectricity caused by the applied static compressive strain would further improve the photoresponse performance. Consequently, under a forward bias of +1 V, compared to the Si/ZnO 2D NSs heterojunction PD without ferroelectricity under strain‐free condition, the photoresponsivity *R* of the PD with ferroelectricity are improved from 35.1 to 120.3 mA W^−1^ under compressive strain with a 2.4‐fold increase, accompanied with faster response speed. Our results present the feasibility of coupling the ferroelectricity with the piezo‐phototronic effect in 2D ferroelectric material to enhance the photoresponse behavior, which offers a good route to enable the development of high‐performance electronic and optoelectronic devices.

## Experimental Section

*Device Fabrication Process*: First, (001) p‐Si wafers (1–10 Ω cm, B‐doped, Universal Wafer) were etched in 5 wt% KOH at 80 °C for 60 min and then were cleaned with acetone, deionized water, and isopropyl alcohol as substrates.^37^ After then, 0.04 m zinc acetate dehydrate dissolved in ethanol as ZnO seed solution was spin coated on the etched Si wafer substrates at 1200 rpm for 1 min and heated at 140 °C for 10 min in air. The coating and heating process was repeated for six times and was placed into an oven at 200 °C for 1 h in air. Next, the etched Si wafer substrate with ZnO seed layer was placed into a glass container with the growth surface facing down, and then a mixture solution with 25 × 10^−3^ zinc nitrate hexahydrate, 25 × 10^−3^ hexamethylenetetramine, and 1 × 10^−3^ V_2_O_5_ was added to the container for the growth of V‐doped ZnO NSs, which occurred at 95 °C for 2 h.^9^ For the growth of ZnO 2D NSs, a layer of Al with a thickness of 10–20 nm was deposited on the etched Si wafer as seed layer, and then the substrate was immersed into a aqueous solution with 25 × 10^−3^ zinc nitrate hexahydrate and 25 × 10^−3^ hexamethylenetetramine, and heated at 95 °C for 2 h. Then, at room temperature a layer of indium tin oxide top electrode (40 Ω ◻^−1^) was deposited on ZnO NSs and a layer of aluminum layer bottom electrode (3.6 Ω ◻^−1^) was deposited on the Si substrate by radio frequency magnetron sputtering (physical vapor deposition (PVD) 75, Kurt. J. Lesker Company) at the power of 120 W and the chamber pressure of 8 mTorr for 30 min, respectively. Finally, a layer of polydimethylsiloxane was utilized to package the devices.

*Characterization and Measurement*: Detailed morphologies and structures of the etched Si and ZnO NSs were characterized by scanning electron microscopy (Hitachi SU8010) with EDX and transmission electron microscopy (Tecnai G2) with SAED. By using an UV–vis spectrophotometer (JΛSCO V‐630), transmission spectrum of ZnO NSs was obtained. The ferroelectric polarization hysteresis loops were conducted using a ferroelectric analyzer (TF Analyzer 2000, Aixacct, Germany) at 1 Hz. Current–voltage and Current–time characteristics were recorded using a computer‐controlled measurement system consisted of a low noise current preamplifier (Model No. SR570, Stanford research systems (SRS)), a low noise voltage preamplifier (Model No. SR560, SRS), and a general‐purpose interface bus (GPIB) controller (GPIB‐USB‐HS, NI 488.2). The 442 nm laser and 1064 nm laser were provided by a He‐Cd dual‐color laser (KI5751I‐G, Kimmon Koha Co., Ltd.) and a fiber coupled laser source (MCLS1, Thorlabs Inc.), respectively. A thermopile powermeter (Newport 1919‐R) was used to measure the light power density.

## Conflict of Interest

The authors declare no conflict of interest.

## Supporting information

Supplementary
