# Supplementary material for: Ferroelectricity‐Enhanced Piezo‐Phototronic Effect in 2D V‐Doped ZnO Nanosheets
Source: Adv Sci (Weinh). 2019 Jun 22;6(16):1900314. doi: 10.1002/advs.201900314 (PMC6702758; doi:10.1002/advs.201900314)
Supplement: Supplementary file 1 — Supplementary [file ADVS-6-1900314-s001.pdf]

Copyright WILEY-VCH Verlag GmbH & Co. KGaA, 69469 Weinheim, Germany, 2019.

ADVANCED SCIENCE Open Access

Supporting Information

for *Adv. Sci.,* DOI: 10.1002/advs.201900314

Ferroelectricity-Enhanced Piezo-Phototronic Effect in 2D V-Doped ZnO Nanosheets

*Yejing Dai, Changsheng Wu, Zhiyi Wu, Zhihao Zhao, Li Li, Yang Lu, and Zhong Lin Wang**

Supporting Information

Ferroelectricity-Enhanced Piezo-Phototronic Effect in 2D V-Doped ZnO Nanosheets

Yejing Dai, Changsheng Wu, Zhiyi Wu, Zhihao Zhao, Li Li, Yang Lu and Zhong Lin Wang[*]

Dr. Y. Dai, Dr. Z. Zhao

School of Materials, Sun Yat-sen University, Guangzhou 510275, China

Dr. Y. Dai, C. Wu, Dr. Z. Wu, Prof. Z. L. Wang

School of Materials Science and Engineering, Georgia Institute of Technology, Atlanta, GA 30332-0245, USA

E-mail: zhong.wang@mse.gatech.edu

Dr. Y. Dai, Dr. Z. Zhao, L. Li, Y. Lu

Key Laboratory of Advanced Ceramics and Machining Technology, Ministry of Education, School of Materials Science and Engineering, Tianjin University, Tianjin 300072, China

Prof. Z. L. Wang

Beijing Institute of Nanoenergy and Nanosystems, Chinese Academy of Sciences, Beijing 100083, China

Content:

Figures S1-S7

Table S1

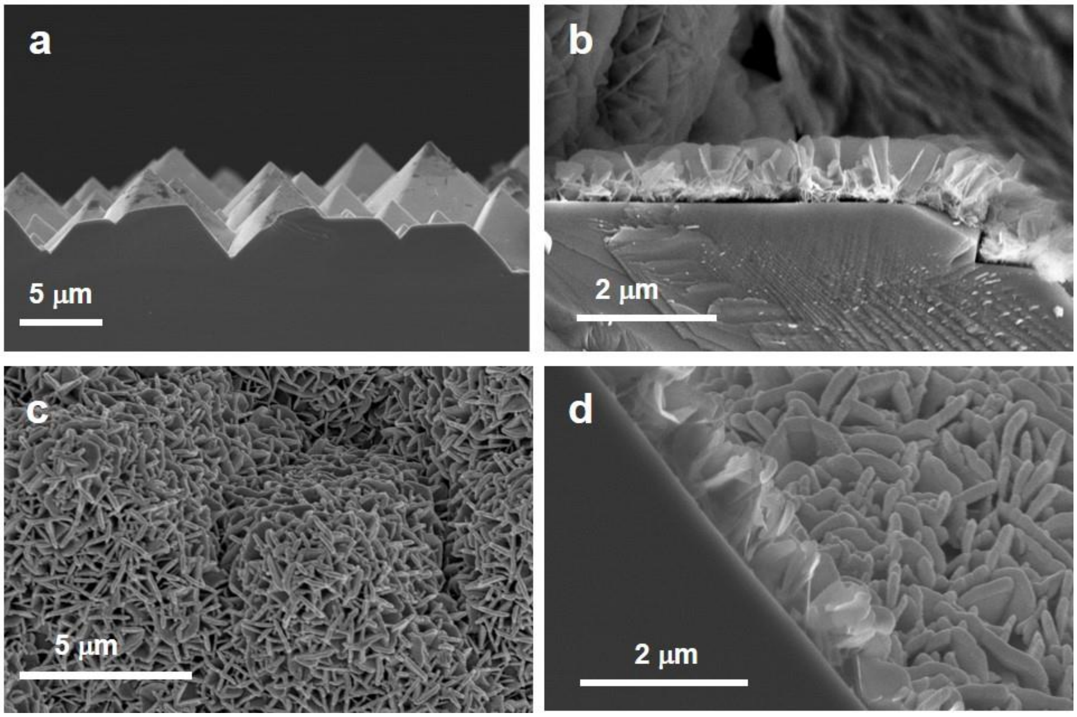

Figure S1. SEM images of a) the etched Si section, b) the V-doped ZnO 2D NSs grown on the etched Si surface, and c, d) the V-doped ZnO 2D NSs after ITO coating.

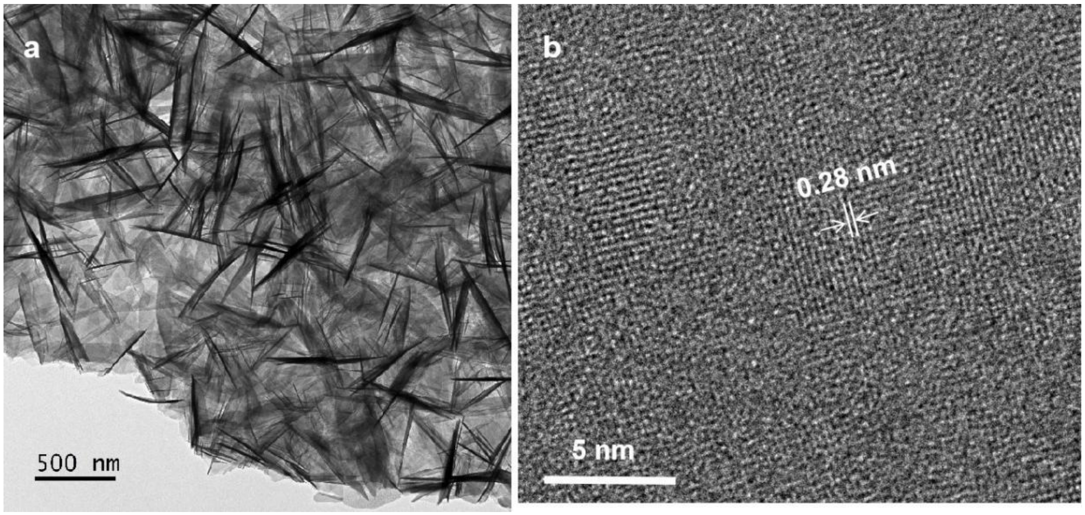

Figure S2. TEM image of the V-doped ZnO 2D NSs.

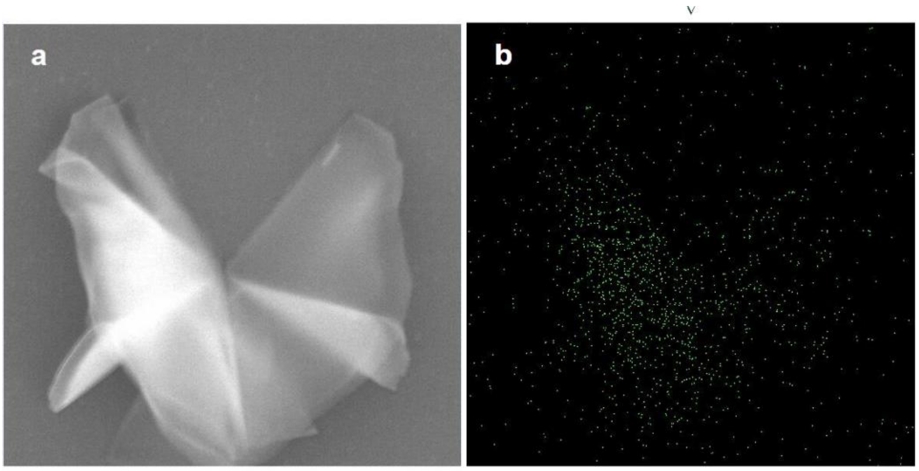

Figure S3. a) SEM image of V-doped ZnO 2D NSs and b) corresponding EDX scanning elemental mappings of V element.

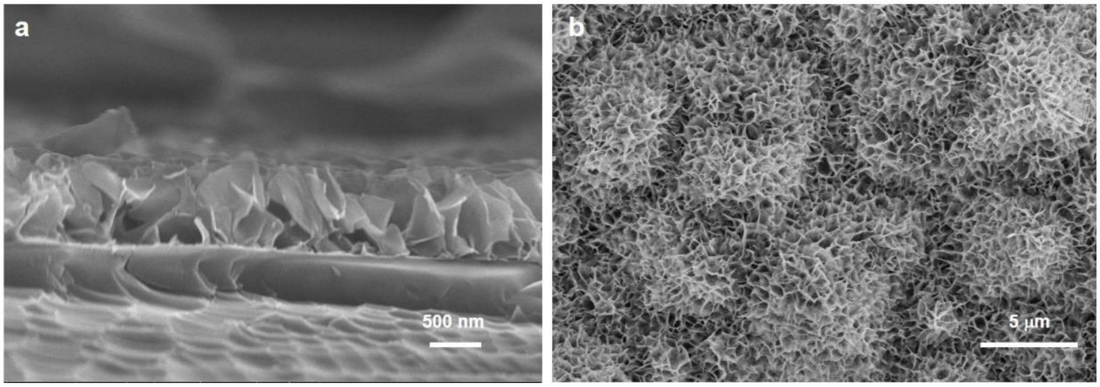

Figure S4. SEM images of non-ferroelectric ZnO 2D NSs: a) section and b) surface.

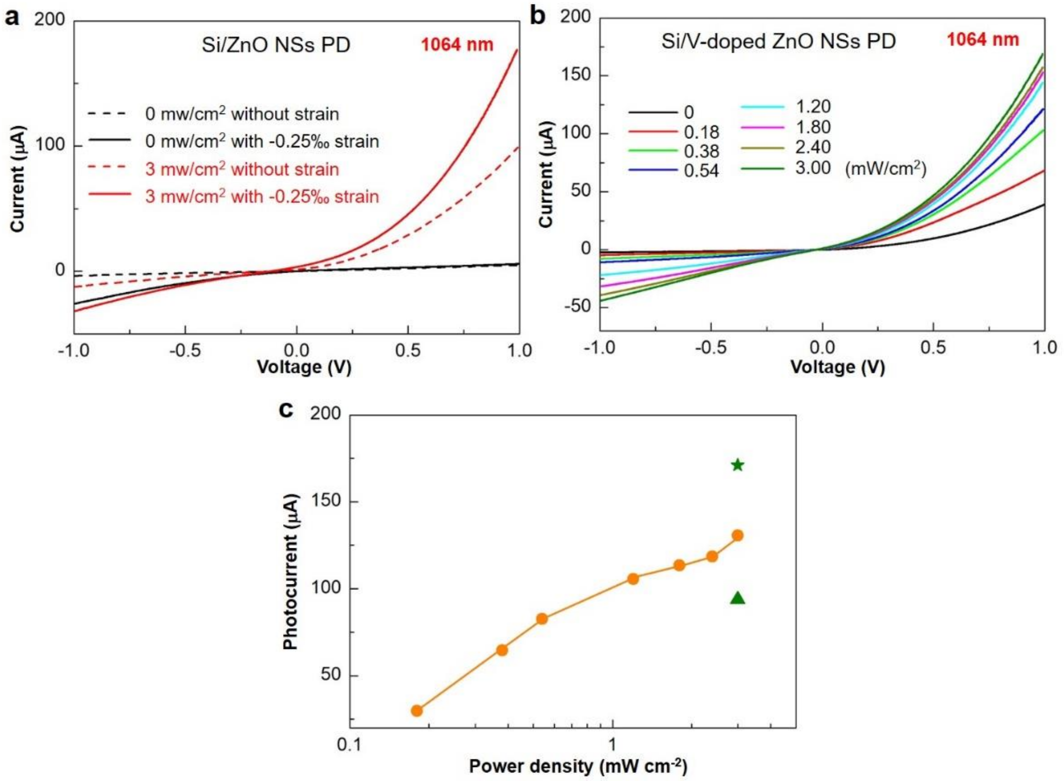

Figure S5. Improved photoresponse performance by the ferroelectricity. a) *I*-*V* characteristics of the SZ-PD without (dotted line) and with (straight line) a -0.25‰ strain

under dark and light illumination (1064 nm at a power density of 3 mW/cm^{-2}) conditions when a 1 V bias is applied. b) *I*-*V* characteristics of the FESZ-PD under different 1064 nm light illumination power densities when a 1 V bias is applied. c) Photocurrent changes with the power density at +1 V forward bias (orange ball is for FESZ-PD, and green triangle and green star are for SZ-PD without and with -0.25‰ strain, respectively).

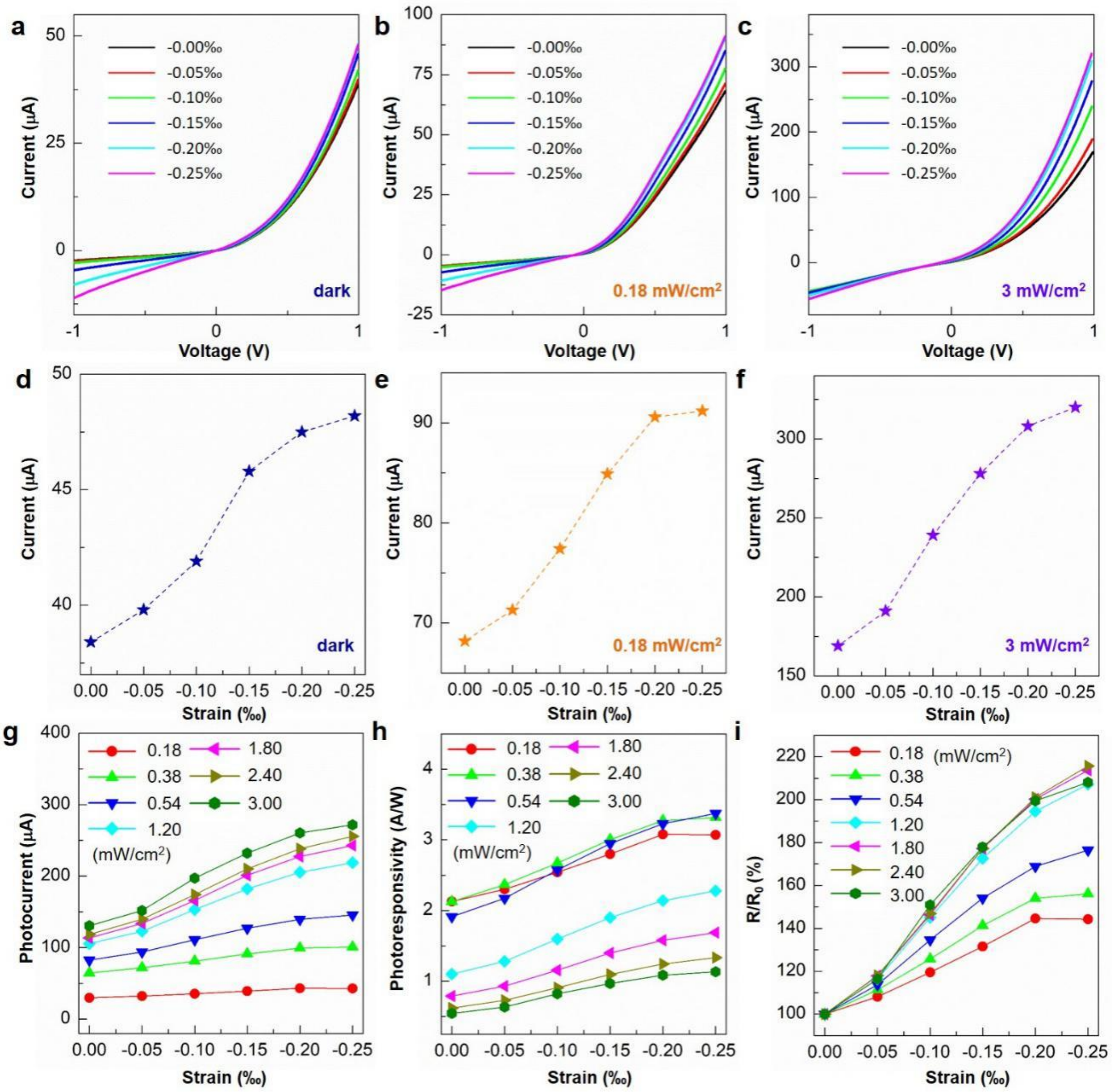

Figure S6. The piezo-phototronic effect on the FESZ-PD. a-c) *I*-*V* characteristics of the device under different compressive strain and 1064 nm light illumination conditions when a 1 V bias is applied. d-f) Output currents of the device under different compressive strain and light illumination conditions when the forward bias is +1 V. g-i) Strain dependence of photocurrent (g), photoresponsivity (h), and R/R_0 (i) for the device under

different light illumination conditions when the forward bias is +1 V.

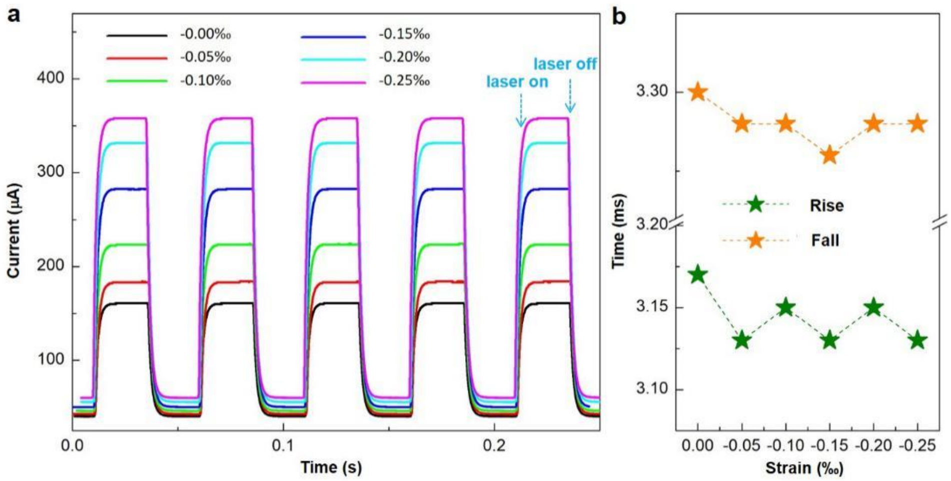

Figure S7. Repeatability and response speed of the FESZ-PD. a) Transient *I*–*t* characteristics of the FESZ-PD under 3 mW/cm^{-2} 1064 nm light illumination and different compressive strains when the forward bias is +1 V. b) Corresponding rise and fall times of the heterojunction PD under 3 mW/cm^{-2} 1064 nm light illumination and different compressive strains.

Table S1. A comparison of photoresponse properties for various nanostructure photodetectors.

Material	Device type	Wavelength (nm)	Bias (V)	*R* (A/W)	Rise/Fall time (ms)	Reference
ZnO NSs/p-Si	p-n junction	442 (10 mW/cm^2)<br>1064 (0.54 mW/cm^2)	+1	0.12<br>3.45	3.07/3.22<br>3.13/3.27	This work
ZnO nanowires/p-Si*	p-n junction	442 (10 mW/cm^2)<br>1060 (0.5 mW/cm^2)	-2	~0.12<br>~0.35	0.97/1.30<br>0.84/1.28	[1]
ZnO nanowires/p-Si*	p-n junction	405 (6 mW/cm^2)	-1	~0.035	154/41	[2]
TiO_2 nanorods/p-Si	p-n junction	440	+5	<0.07	–	[3]
ZnO/Si branched nanowires	p-n junction	440<br>1060	-1	~0.006<br><0.004	–	[4]
CdS nanowires/p-Si*	p-n junction	1064 (0.46 mW/cm^2)	+1.5	0.013	>100/>50	[5]
CdS nanowires/p-Si	p-n junction	1100	-1	~1	–	[6]
graphene/Si	Schottky Junction	850	–	0.029	~0.1	[7]
ZnO nanorods	M-S	450	–	–	3700/60000	[8]

*The data for these PD devices are under strain-free condition for comparison.

[1] H. Zou, X. Li, W. Peng, W. Wu, R. Yu, C. Wu, W. Ding, F. Hu, R. Liu, Y. Zi, Z. L. Wang, *Adv. Mater.* **2017**, *29*, 1701412.

[2] Z. Pan, W. Peng, F. Li, Y. He, *Adv. Funct. Mater.* **2018**, *28*, 1706897.

[3] A. M. Selman, Z. Hassan, M. Husham, N. M. Ahmed, *Appl. Surf. Sci.* **2014**, *305*, 445.

[4] K. Sun, Y. Jing, N. Park, C. Li, Y. Bando, D. Wang, *J. Am. Chem. Soc.* **2010**, *132*, 15465.

[5] Y. Dai, X. Wang, W. Peng, C. Wu, Y. Ding, K. Dong, Z. L. Wang, *Nano Energy* **2018**, *44*, 311.

[6] S. Manna, S. Das, S. P. Mondal, R. Singha, S. K. Ray, *J. Phys. Chem. C* **2012**, *116*, 7126.

[7] P. Lv, X. J. Zhang, X. W. Zhang, W. Deng, J. S. Jie, *IEEE Electr. Device L.* **2013**, *34*, 1337.

[8] S. E. Ahn, J. S. Lee, H. Kim, S. Kim, B. H. Kang, K. H. Kim, G. T. Kim, P *Appl. Phys. Lett.* **2004**, *84*, 5022.
